# Development, characterization and antimicrobial activity and wound healing nanocomposite membranes xanthan: silver using porcine model

**DOI:** 10.1186/1753-6561-8-S4-P218

**Published:** 2014-10-01

**Authors:** Klebson Silva Santos, Malone Santos Pinheiro, Andriele Mendonça Barbosa, Eleci Adriano Hendges, Clauberto Rodrigues de Oliveira, Paula Santos Nunes, Madson Rodrigo Silva Bezerra, Adriely Maria Oliveira Rocha, Peterson Gomes dos Santos, Juliana Cordeiro Cardoso, Ricardo Luiz Cavalcanti de Albuquerque Júnior, Francine Ferreira Padilha

**Affiliations:** 1 Programa de Doutorado da Rede Nordeste de Biotecnologia, Universidade Tiradentes, Murilo Dantas, 300, Farolândia, CEP 49.032-490, Aracaju, SE, Brazil; 2 Programa de Pós, Graduação em Biotecnologia Industrial, Universidade Tiradentes, Avenida Murilo Dantas, 300, Farolândia, CEP 49.032-490, Aracaju, SE, Brazil; 3 Laboratório de Biomateriais, Instituto de Tecnologia e Pesquisa/ Universidade Tiradentes, Avenida Murilo Dantas, 300, Farolândia, CEP 49.032-490, Aracaju, SE, Brazil

## Background

In recent years, advances in biotechnology has allowed the development of synthetic membranes associated with nanocomposites, which has shown promising results as dermal burns dressings. In this sense, silver nanoparticles (NPAg) has been the focus of interest because of their biological properties such as antimicrobial and anti-inflammatory effect. The incorporation of NPAg in biological membranes of different natures, such as chitosan, polyester, polymethacrylate methyl and cellulose, has been successfully tested in several biological models. The association between NPAg and polymers produced by the micro-organism presents important advantages, such as water solubility and lack of toxicity. Recently we developed a technique for producing NPAg associated with xanthan (GX), a biopolymer with potential application in various sectors of the petrochemical industry, food and pharmaceutical, through fermentation by *Xanthomonas *sp performed in the presence of silver nitrate.

## Methods

Therefore, this study aimed to develop, characterize and evaluate the potential antimicrobial and healing membranes nanocomposite xanthan: silver on second-degree burns in the porcine model. Therefore, xanthan biocomposites: silver were used for fabrication of membranes (for casting process, which were subsequently characterized for thickness, mechanical properties (stress, strain, Young's modulus) and the thermal profile (DSC, TG and DTG). Activity antimicrobial was tested against strains of *Escherichia coli *(ATCC 25922) and *Staphylococcus aureus *(ATCC 25923). analysis for tissue repair were made two dermal burns on the back nine male pigs breed Yorkshire (25 ± 5 kg), treated with Xanthan biosensor membrane: silver (XNPAg) with topical application of silver sulfadiazine 1% (SDZ).

## Results and conclusions

After eight, 18 and 30 days the wounds were examined macroscopically determined for each lesion area, and the animals euthanized for Microscopic study of the scar area observed that XNPAg membranes showed a significant increase in the values of thickness (P <0.05), density (p <0.01) and Young's modulus (p <0.001) and reduced strength strain (p <0.05) when compared to membranes of xanthan. Were revealed changes in the thermal profile of the two membranes suggesting the incorporation of silver nanoparticles in the polymer xanthan. XNPAg The membrane induced the formation of inhibition zones 9, 7 mm and 9.6 mm and death rate of 89% and 100% for *Staphylococcus aureus *and *Escherichia coli *respectively. Histological analysis showed quantitative and qualitative increase in the reaction granulation and best architectural arrangement of collagen fibers along the the healing process of wounds covered with membranes XNPAg. Could be concluded that the membranes nanocomposite xanthan:silver showed satisfactory mechanical properties for its handling, transportation and storage, as well as important antimicrobial activity and pro-healing in dermal burns using porcine model.
